# Community health worker-led versus facility-based type 2 diabetes care in rural Lesotho: a cluster-randomized trial within the ComBaCaL cohort study

**DOI:** 10.1186/s12916-026-04943-4

**Published:** 2026-05-22

**Authors:** Felix Gerber, Ravi Gupta, Giuliana Sanchez-Samaniego, Thabo Ishmael Lejone, Thesar Tahirsylaj, Fabian Raeber, Elis Belen Saavedra, Iliana Esquivel-Valdés, Mamakhala Chitja, Manthabiseng Molulela, Makhebe Khomolishoele, Mota Mota, Matumaole Bane, Sesale Masike, Mamorontsáne Pauline Sematle, Manthati Mofokeng, Retselisitsoe Makabateng, Madavida Mphunyane, Lebohang Sao, Mosa Tlahali, Malitaba Litaba, Dave Brian Basler, Kevin Kindler, Irene Ayakaka, Pauline Grimm, Eleonora Seelig, Frédérique Chammartin, Niklaus Daniel Labhardt, Alain Amstutz

**Affiliations:** 1 Division of Clinical Epidemiology, Department of Clinical Research, University Hospital Basel, Basel, Switzerland; 2 University of Basel, Basel, Switzerland; 3 University Department of Medicine, Division of General Internal and Emergency Medicine, Kantonsspital Aarau, Aarau, Switzerland; 4 SolidarMed Lesotho, Maseru, Lesotho; 5 Ministry of Health Lesotho, Maseru, Lesotho; 6 SolidarMed Switzerland, Lucerne, Switzerland; 7 Clinic of Endocrinology, Diabetology and Metabolism, University Hospital Basel, Basel, Switzerland; 8 Oslo Center for Biostatistics and Epidemiology, Oslo University Hospital, University of Oslo, Oslo, Norway; 9 Electronic Health Records Group, Population Health Sciences, Bristol Medical School, University of Bristol, Bristol, United Kingdom

**Keywords:** Diabetes, Metformin, Prescription, Community Health Workers, Africa, Lesotho, Access to care, Differentiated service delivery, Clinical decision support systems

## Abstract

**Background:**

Type 2 diabetes poses a growing public health burden in low- and middle-income countries, where major gaps in access to chronic care persist. Task-shifting to Community Health Workers (CHWs) and the use of digital clinical decision support systems may bridge these gaps. Randomized trials evaluating CHW-led care models in which CHWs initiate, titrate, and monitor first-line diabetes treatment are lacking.

**Methods:**

We conducted a cluster-randomised trial nested within the ComBaCaL (Community-Based Chronic Care Lesotho) cohort (NCT05596773). The cohort spans 103 rural villages in Lesotho, managed by trained, supervised CHWs. After home-based screening of cohort participants ≥40 years old or with body mass index ≥25 kg/m^2^, all with type 2 diabetes were enrolled in the trial. In intervention villages, CHWs - guided by a tablet-based clinical decision support system - provided care, including initiation and monitoring of metformin, atorvastatin and aspirin. In control villages, participants were referred to facility-based care. The primary analysis included participants with uncomplicated and uncontrolled type 2 diabetes (fasting glucose ≥7 mmol/l) on glycated haemoglobin (HbA1c) at 12 months.

**Results:**

From 13 May 2023 to 31 January 2024, 5’785 cohort participants were screened, 252 (4·4%) diagnosed with type 2 diabetes, and 103 (51 control, 52 intervention) included in the primary analysis (73·8% female, mean age 62·3 ± 12·8 years, mean HbA1c 7·2 ± 1·4%). At 12 months, HbA1c was 7·1 ± 1·9% in the control arm and 6·5 ± 1·3% in the intervention arm (adjusted mean difference −0·46%, 95%CI −1·14 to 0·22). Engagement in care was higher in the intervention arm. No relevant difference in safety outcomes was observed.

**Conclusions:**

CHW-led, clinical decision support system-assisted management of type 2 diabetes, including first-line drug prescription, may improve engagement in care and glycaemic control in rural low-resource settings. Larger studies are required to confirm these findings.

**Trial registration:**

Clinicaltrials.gov: NCT05743387

## Background

In 2022, 828 million adults worldwide were living with diabetes, a fourfold increase since 1990, with 80% residing in low- and middle-income countries [1, 2]. Less than half of the people living with diabetes in low- and middle-income countries receive treatment [1]. Compared with high-income countries, people living in low- and middle-income countries face a substantially higher risk of remaining undiagnosed, untreated, or poorly controlled, and of developing early complications, particularly in rural areas where access to care is challenging [2–5].

Expanding access to quality diabetes care requires scalable models of care that enable effective service delivery in both urban and rural settings [6]. Given the severe shortages of healthcare professionals in low- and middle-income countries, task-shifting to Community Health Workers (CHWs) - lay people recruited from the community they serve - offers a potential solution, and is promoted by the World Health Organisation (WHO) [7, 8]. Care models whereby CHWs offer specific aspects of prevention and care have been shown to be feasible, safe, and effective at improving access to healthcare services in their communities across multiple disease areas [9, 10]. Previous CHW-led diabetes interventions have typically included education, lifestyle counselling, screening, referral and self-management support [8, 11–15]. However, a key limitation of all prior CHW-led diabetes intervention evidence is the restricted scope of CHW responsibilities, especially excluding prescribing authority for first-line pharmacotherapy [16].

Digital clinical decision support systems (CDSS) assist clinical decision-making by providing personalized assessments and recommendations based on individual patient characteristics [17]. By offering algorithmic guidance and facilitating remote supervision, CDSS may enable CHWs to provide more complex services, including prescription of medication, safely and efficiently [18–21]. Allowing CHWs guided by a CDSS to independently initiate, monitor and titrate first-line pharmacotherapy at patients’ homes - in addition to screening, education, counselling and referral services - may unlock the full potential of CHW-led care models in improving access to and clinical outcomes of diabetes care. We are not aware of any randomized trial that evaluated such a diabetes care model in low- and middle income countries.

This intervention builds on the assumption that limited access to facility-based chronic disease services represents a key barrier to effective diabetes management in rural settings. By enabling CHWs to initiate and monitor first-line pharmacological treatment at the community level, supported by a digital clinical decision support system, the intervention aims to improve access to treatment and continuity of care. The hypothesized mechanism of effect is that improved access to treatment increases linkage to care and sustained engagement in care, which in turn contributes to improved glycaemic control and cardiovascular risk management.

This cluster-randomized trial aimed to assess the effectiveness of a CHW-led, CDSS-assisted, diabetes care model including independent metformin, atorvastatin and aspirin initiation, titration and monitoring in rural Lesotho.

## Methods

### Study design and setting

We conducted a 1:1 cluster-randomized superiority trial nested within the Community Based chronic Care Lesotho (ComBaCaL; NCT05596773) cohort study following a Trials within Cohorts (TwiCs) design [22]. The cohort spans 103 randomly selected rural villages in the two districts Butha-Buthe and Mokhotlong in Northeastern Lesotho. It is embedded within the government-led community health programme, managed by local CHWs, and serves as a platform to investigate chronic diseases and their management [23].

Lesotho is a representative example of an African lower-middle-income country where the developing health system is facing a dual burden of persistently high-prevalence infectious diseases (HIV/AIDS and tuberculosis) and a rapidly increasing epidemic of non-communicable diseases (NCDs) [2, 24]. CHWs play an important role in linking the community to facility-based health services and have effectively contributed to the improved control of HIV/AIDS, especially in remote rural areas [25–27].

The intervention described here has been developed in collaboration with local community members and the Lesotho Ministry of Health based on a local NCDs prevalence survey and burden assessment [24, 28], a scoping literature review [29], and a pilot study [30] in the same area.

The study protocol has been published [31], and the trial is registered on clinicaltrials.gov (NCT05743387), where the full protocol and statistical analysis plan are available. This study is reported according to the Consolidated Standards of Reporting Trials (CONSORT) cluster randomized trial extension [32], with the checklist provided in the Additional File 1: Table S1.

### Participants

CHWs screened all adult ComBaCaL cohort participants with a body-mass index (BMI) of ≥25 kg/m^2^ or aged ≥40 years for type 2 diabetes via capillary blood glucose measurements according to a diagnostic algorithm based on the Lesotho Standard Treatment Guidelines [33]. All non-pregnant cohort participants with type 2 diabetes were enrolled into the trial. Type 2 diabetes was defined as (1) self-reported use of antidiabetic medication; (2) a fasting blood glucose (FBG) ≥ 7 mmol/l or a random blood glucose (RBG) ≥ 11.1 mmol/l after a previously elevated FBG or RBG ≥ 5.6 mmol/l; or 3) an FBG ≥ 7 mmol/l or an RBG ≥ 11.1 in presence of all three cardinal symptoms of uncontrolled diabetes (polyuria, polydipsia, and weight loss) in the absence of a clinical history suggestive of type 1 diabetes. Among all trial participants, those with uncomplicated, uncontrolled diabetes were included in the pre-specified primary analysis population (primary analysis set). Uncontrolled diabetes was defined as baseline FBG ≥ 7.0 mmol/l and uncomplicated diabetes as taking no antidiabetic treatment or only one oral antidiabetic medication and not requiring referral at enrolment. Referral was indicated in case of RBG > 16.7 mmol/l, FBG > 14.0 mmol/l, or presence of all three cardinal diabetes symptoms.

### Randomization and masking

Half of the ComBaCaL cohort villages were randomly allocated to the intervention arm by a statistician not involved in the study, before the start of screening. The randomization was stratified by district (Butha-Buthe versus Mokhotlong) and access to a health facility (easy versus difficult access, defined as needing to cross a mountain or river or travel >10 km to the nearest health facility). CHWs enrolled participants, provided the intervention, and collected secondary endpoint data. Thus, they were not masked to the intervention. The primary endpoint (glycated haemoglobin [HbA1c]) was measured by study staff not involved in the delivery of the intervention. In line with the TwiCs design, participants in control villages were not informed about the intervention offered in other villages.

### Procedures

Each village had one CHW, equipped with a tailored tablet-based CDSS, providing algorithmic guidance for all study procedures, including data collection and service delivery. In line with Lesotho’s Ministry of Health Village Health Programme policy [27], CHWs were elected by their communities and linked to their catchment health facility, where they met monthly and could seek additional guidance from facility nurses. Supervising study team members monitored CHW activities through a web-based dashboard, and CHWs could request support as needed. All CHWs received five days of training on data collection and diabetes screening; intervention CHWs received an additional two-day training. CHWs were paid the regular government stipend (40$/month) plus compensation for study-related work (20$/month), with no additional remuneration for CHWs in intervention villages.

In intervention villages, CHWs offered a community-based diabetes care package including lifestyle counselling, first-line antidiabetic medication (metformin), lipid-lowering therapy (atorvastatin 10 mg) and antiplatelet therapy (aspirin 100 mg for those with a previous myocardial infarction or stroke), based on the Lesotho Standard Treatment Guidelines [33], and guided by the CDSS application. Participants not taking metformin already and not meeting any referral criteria were offered metformin initiation at 500 mg/day with weekly titration to the maximum dose of 2 g/day. In case of FBG or RBG < 4.4 mmol/l, the metformin dose was reduced by 500 mg/day. For participants with complicated diabetes or fulfilling referral criteria, the CDSS prompted CHWs to consult a healthcare professional to guide treatment initiation. Participants with FBG > 8.0 mmol/l or RBG > 9.9 mmol/l and reporting good adherence to the maximum dose of metformin were referred to the closest health facility for further management. In summary, the intervention allowed CHWs, guided by the CDSS, to independently monitor diabetes treatment,to initiate and titrate metformin treatment for eligible participants, and to determine referral of more complex participants to healthcare professionals. All participants were eligible for anti-lipid treatment due to the increased cardiovascular risk associated with diabetes. The detailed treatment algorithm is provided in the Additional File 2: Fig. S1. Services were voluntary; those declining CHW-led care were referred to the health facility but remained under two-monthly CHW follow-up at the community level. All medication provided followed the routine delivery pathways through the National Drug Service Organisation of Lesotho to the health facilities. CHWs restocked medication during their routine monthly health facility gatherings.

In control villages, CHWs used the same tablet-based CDSS for data collection but only provided lifestyle counselling and referred participants to the health facility for pharmacological diabetes management with follow-up and re-referral after six months if indicated.

### Outcomes

The primary endpoint was HbA1c twelve months after enrolment, assessed on capillary blood by the study team using A1CNow+ (PTS Diagnostics, USA). Secondary endpoints included HbA1c six months after enrolment and the following after six and twelve months: linkage to care (initiating antidiabetic treatment among those untreated at enrolment), engagement in care (reporting antidiabetic medication intake or reaching treatment targets without medication), self-reported adherence (adherent if reporting intake of antidiabetic medication on all of the last four days), HbA1c < 8%, FBG < 7 mmol/l, use of lipid-lowering medication, 10-year cardiovascular risk (WHO lab-based prediction tool [34]), body-mass index (BMI), abdominal circumference, lipid profile (total cholesterol, low-density lipoprotein [LDL], triglycerides, total cholesterol to high-density lipoprotein [HDL] ratio), self-reported physical activity (using the International Physical Activity Questionnaire Short Form [IPAQ-SF] [35]), as well as alcohol and tobacco use. Safety outcomes were the occurrence of serious adverse events and of adverse events of special interest which were defined as adverse events consistent with diabetes complications, such as stroke, myocardial infarction, hyperglycaemic events (RBG > 16.7 mmol/l or FBG > 14.0 mmol/l or presence of all three cardinal symptoms of diabetes), new diagnosis of heart failure, chronic kidney disease, blindness, diabetic foot syndrome and adverse events probably related to the intake of antidiabetic medication, such as hypoglycaemia (BG < 3 mmol/l with symptoms of hypoglycaemia) and or other side effects leading to treatment discontinuation of the medication concerned. A 150–240 day window was applied for six months endpoints, and a 300–420 day window for twelve months endpoints.

HbA1c was collected by study staff, while all other outcomes were collected by CHWs. During follow-up visits, CHWs in both arms actively inquired about the occurrence of possible adverse events, but also passively solicited adverse events via participants, friends, relatives and health facility staff reports, as well as screening of participants’ “bukanas” (personal health booklets). Adverse events flagged by the CHWs were verified by supervising study staff and classified by a blinded physician.

Further details are outlined in the protocol [31] and the statistical analysis plan available on clinicaltrials.gov (NCT05743387).

### Data management, sample size and statistical analysis

Each CHW received a password protected tablet with the CDSS application installed. The application was based on the Community Health Toolkit Core Framework, a widely-used, offline-first, open-source software designed for community health systems [36]. Data were synchronized regularly to a secured server and monitored locally and centrally.

An a priori sample size calculation was powered for the primary analysis population (uncomplicated [no treatment or only one oral antidiabetic medication at baseline], uncontrolled [baseline FBG ≥ 7 mmol/l] diabetes), and calculated for a corresponding individually-randomized trial inflated by the standard cluster design effect to account for within-cluster correlation [37]. Based on a recent NCD prevalence survey in Lesotho [24], adult diabetes prevalence in the rural setting in Lesotho was estimated at 4%, with 60% fulfilling the primary analysis population criteria. With an average village size of 100 adult inhabitants, we expected mean 2.4 eligible participants per village for the primary analysis. Assuming a clinically relevant HbA1c difference of 0.6% [38] between the two arms after 12 months, an intra-cluster correlation of 0.015, and 20% attrition, 240 individuals (120 per arm, 50 clusters per arm) were required to detect superiority with a type I error of 0.05 and a statistical power of 80%.

The primary analysis included all participants as randomized but excluded participants who experienced the intercurrent events of death, pregnancy, or relocation outside the study area during follow-up (principal stratum strategy). This approach defines the estimand on participants that did not experience intercurrent events, allowing a meaningful assessment of the effect of our intervention in participants for whom the outcome could be observed.

For the primary outcome, we used a linear mixed-effects regression model adjusted for stratification factors district (Mokhotlong versus Butha-Buthe), access to health facilities (easy versus hard, defined as needing to travel more than 10 kilometers, to cross a mountain or river to reach the closest health facility), self-reported sex (female versus male), age (continuous), and baseline HbA1c levels (continuous), with a random intercept at the cluster level. HbA1c values for participants lost to follow-up or withdrawing consent were imputed under a missing-at-random assumption using a linear model fitted on baseline FBG (missing; low <5.6 mmol/l; moderate 5.6–7.0 mmol/l; high ≥7.0 mmol/l) and baseline characteristics as pre-specified in the statistical analysis plan.

Sensitivity analyses assessed alternative assumptions for intercurrent events, including a hypothetical strategy in which participants who moved away or became pregnant were included through imputation of HbA1c values.

For analyses of secondary outcomes, we used mixed effects logistic or linear regression models adjusted for baseline outcomes in addition to stratification factors, without formal testing.

We calculated adjusted odds ratios (aORs) for binary endpoints and adjusted mean difference (aMD) for continuous endpoints, together with 95% confidence intervals (95%CI). Robustness of the intervention effect on the primary endpoint was assessed for the following predefined factors: age (continuous), sex (female versus male), access to health facility (easy versus hard), diagnosis status (newly diagnosed versus already in care for diabetes). Following the Instrument for assessing the Credibility of Effect Modification Analyses [39], we conducted a credibility assessment in case of a p-value of interaction below 0.1.

We conducted several sensitivity analyses: (a) including all participants enrolled irrespective of treatment or baseline glucose levels (all participants set) which we considered our key sensitivity since it entails all trial participants and hence was displayed across all results; (b) including only participants with uncomplicated, uncontrolled diabetes and baseline HbA1c ≥ 6.5% (strict inclusion set); (c) including only participants with baseline HbA1c ≥ 6.5% irrespective of baseline medication or FBG levels (HbA1c set); (d) including only participants with available baseline and endpoint HbA1c (complete case set); (e) applying a hypothetical strategy instead of a principal stratum strategy in the primary analysis set for participants who moved away or became pregnant using the same HbA1c imputation model as described above (hypothetical estimand).

Participants were free to accept or reject any of the offered intervention services, which precludes defining an individual-level protocol deviation in a trial with a cluster-level intervention. Hence, we did not conduct a per-protocol analysis.

No interim analysis was conducted. Further details are outlined in the protocol [31] and the statistical analysis plan. Data management was done using Stata IC version 16.0 and data analysis using R version 4.3.3.

## Results

**Fig. 1:**
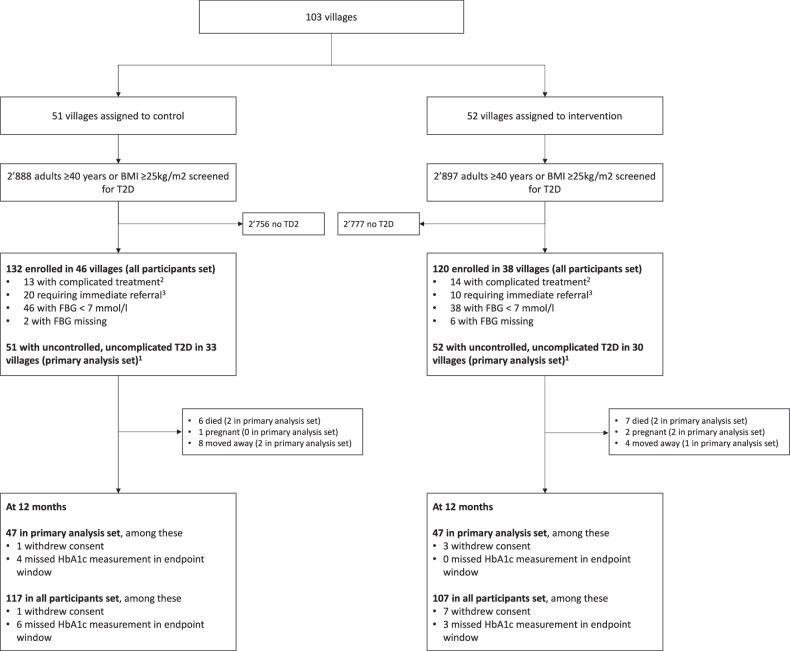
Consort Flow Diagram. 1: Uncomplicated (no treatment or only one oral antidiabetic medication), uncontrolled (FBG ≥ 7 mmol/l). 2: Complicated treatment: insulin or more than one oral antidiabetic medication. 3: Immediate referral indicated if FBG > 14 mmol/l or random blood glucose >16.7 mmol/l or all three cardinal diabetes symptoms (polyuria, polydipsia, weight loss). Abbreviations: T2D type 2 diabetes, FBG fasting blood glucose, BMI body-mass-index

From May 13, 2023, to January 31, 2024, CHWs screened 5’785 ComBaCaL cohort participants aged ≥40 years or having a BMI ≥ 25 kg/m^2^ across the 103 randomly sampled and subsequently randomized ComBaCaL cohort villages (Fig. 1). 252 cohort participants (132 across 46 control clusters and 120 across 38 intervention clusters) were identified living with type 2 diabetes and enrolled. Out of those, 103 (51 across 33 control clusters and 52 across 30 intervention clusters) had uncomplicated, uncontrolled diabetes (primary analysis set).

Enrolment was stopped after the screening across the entire ComBaCaL cohort reached near full coverage. This resulted in half of the anticipated a priori calculated sample size for this trial. Further recruitment was not possible since this would have required to expand the entire cohort study. Because the achieved sample size was substantially smaller than anticipated, the trial was underpowered for formal superiority testing. We therefore report effect sizes with 95% confidence intervals and interpret findings cautiously.

Table 1 presents the main baseline characteristics, across the all participants set and the primary analysis set. Participants of the primary analysis set had a mean age of 62.3 years (standard deviation (SD) 12.8), a mean HbA1c of 7.2% (SD 1.4), a mean FBG of 8.8 mmol/l (SD 1.7), and a mean BMI of 30.0 kg/m^2^ (SD 5.9). Overall, 73.8% were female; 25.2% were already in care for diabetes at baseline, 20.4% were smokers, and 14.6% reported living with HIV. The proportion of participants already receiving diabetes care at baseline was higher in the intervention arm (34.6%) than in the control arm (15.7%) in the primary analysis set. This imbalance likely reflects random variation in the context of small cluster sizes.

**Table 1:** Baseline characteristics of trial participants by study arm and analysis sets

	Primary analysis set			All participants set		
	Control	Intervention	Total	Control	Intervention	Total
**Cluster level, villages**	n = 33	n = 30	n = 63	n = 46	n = 38	n = 84
Participants per village, median (IQR)	1.0 (1.0, 2.0)	1.0 (1.0, 2.0)	1.0 (1.0, 2.0)	2.0 (1.0, 4.0)	2.0 (1.3, 4.8)	2.0 (1.0, 4.0)
Hard to access health facility^a^, n (%)	18 (54.5)	14 (46.7)	32 (50.8)	25 (54.3)	19 (50.0)	44 (52.4)
Butha Buthe district, n (%)	20 (60.6)	17 (56.7)	37 (56.9)	25 (54.3)	19 (50.0)	44 (52.4)
**Individual-level, participants**	N = 51	N = 52	N = 103	n = 132	n = 120	n = 252
**Demographics and anthropometrics**						
Female, n (%)	34 (66.7)	42 (80.8)	76 (73.8)	101 (76.5)	93 (77.5)	194 (77.0)
Age, years, mean (SD)	61.4 (11.7)	63.2 (14.0)	62.3 (12.8)	61.2 (13.9)	64.0 (12.6)	62.6 (13.3)
Body mass index, kg/m^2^, mean (SD)	29.3 (6.1)	30.6 (5.7)	30.0 (5.9)	29.6 (5.9)	29.9 (5.6)	29.7 (5.8)
Abdominal circumference, cm, mean (SD)	97.0 (13.5)	100.1 (12.9)	98.5 (13.3)	98.6 (12.6)	98.8 (12.1)	98.7 (12.4)
Education: secondary school or higher, n (%)	12 (23.5)	18 (34.6)	30 (29.1)	32 (24.4)	38 (31.7)	70 (27.9)
Working for pay or self-employed, n (%)	21 (41.2)	25 (48.1)	46 (44.7)	47 (35.6)	49 (40.8)	96 (38.1)
Married or in stable relationship, n (%)	30 (58.8)	35 (67.3)	65 (63.1)	76 (58.0)	71 (59.2)	147 (58.3)
**Blood glucose and lipids**						
HbA1c (%), mean (SD)	7.2 (1.5)	7.2 (1.4)	7.2 (1.4)	7.5 (2.0)	7.2 (1.8)	7.3 (1.9)
Fasting blood glucose, mean (SD)	8.7 (1.5)	8.9 (1.9)	8.8 (1.7)	8.6 (3.8)	8.4 (3.2)	8.5 (3.5)
Total cholesterol, mg/dl, mean (SD)	176.9 (42.1)	187.4 (52.9)	182.3 (48.0)	176.8 (46.3)	182.0 (58.6)	179.3 (52.5)
LDL cholesterol, mg/dl, mean (SD)	101.4 (39.2)	112.9 (47.5)	107.7 (44.0)	103.8 (38.8)	107.1 (47.5)	105.4 (43.3)
**Engagement in care indicators and co-conditions**						
Already in care for T2D, n (%)	8 (15.7)	18 (34.6)	26 (25.2)	75 (56.8)	83 (69.2)	158 (62.7)
Already taking a statin, n (%)	0 (0.0)	1 (1.9)	1 (1.0)	4 (4.0)	5 (4.2)	9 (3.6)
Already in care for HIV or hypertension, n (%)	31 (60.8)	38 (73.1)	69 (67.0)	93 (70.5)	97 (80.8)	190 (75.4)
Living with hypertension, n (%)	26 (51.0)	34 (65.4)	60 (58.3)	84 (63.6)	87 (72.5)	171 (67.9)
Living with HIV, n (%)	7 (13.7)	8 (15.4)	15 (14.6)	18 (13.6)	16 (13.3)	34 (13.5)
Current smoking, n (%)	10 (19.6)	11 (21.2)	21 (20.4)	21 (15.9)	18 (15.0)	39 (15.5)
Alcohol consumption ≥1 day/week, n (%)	8 (15.7)	3 (5.8)	11 (10.7)	14 (10.6)	6 (5.0)	20 (8.0)
**CVD related lifestyle factors**						
Low physical activity^b^, n (%)	5 (9.8)	10 (19.6)	15 (14.7)	26 (19.8)	25 (21.2)	51 (20.5)
Sweet food item consumption ≥3 days/week^c^, n (%)	8 (15.7)	9 (17.3)	17 (16.5)	19 (14.5)	24 (20.0)	43 (17.1)
Fried food consumption ≥3 days/week^c^, n (%)	8 (15.7)	9 (17.3)	17 (16.5)	23 (17.6)	24 (20.0)	47 (18.7)
Sweet beverage consumption ≥3 days/week^c^, n (%)	6 (11.8)	7 (13.5)	13 (12.6)	12 (9.2)	14 (11.7)	26 (10.4)

*SD* standard deviation, *LDL* low-density lipoprotein

^a^Needing to cross a mountain or river or travel >10 km to the nearest health facility

^b^Self-reported physical activity using the International Physical Activity Questionnaire Short Form (IPAQ-SF) [35]

^c^Self-reported consumption using a food frequency questionnaire adapted from an assessment tool for obesity used in South Africa [52]

Missing data: HbA1c: 18 in primary analysis set (10 control, 8 intervention) and 34 in all participants set (19 control, 15 intervention); Fasting blood sugar: 8 in all participants set (2 control, 6 intervention); BMI: 3 in primary analysis set (2 control, 1 intervention) and 8 in all participants set (5 control, 3 intervention); Abdominal circumference: 9 in primary analysis set (1 control, 8 intervention) and 23 in all participants set (5 control, 18 intervention); Education status: 1 in all participants set (1 control); Employment status: 1 in all participants set (1 control); Marital status: 1 in all participants set (1 control); Total cholesterol: 14 in primary analysis set (8 control, 6 intervention) and 25 in all participants set (14 control, 11 intervention); LDL cholesterol: 26 in primary analysis set (16 control, 10 intervention) and 56 in all participants set (33 control, 23 intervention); Alcohol consumption: 1 in all participants set (1 control); Physical activity: 1 in primary analysis set (1 intervention), and 3 in all participants set (1 control, 2 intervention); Sweet food: 1 in all participants set (1 control); Fried food: 1 in all participants set (1 control); Sweet beverage: 1 in all participants set (1 control).

**Fig. 2:**
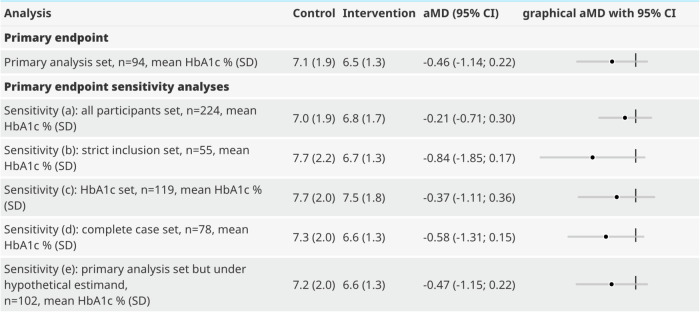
Primary endpoint (HbA1c %, at 12 months) with sensitivity analyses. Abbreviations: SD standard deviation, aMD adjusted mean difference. The intracluster correlation coefficients were as follows from top to bottom 0.11, 0.15, 0.23, 0.18, 0.15, 0.10. The reference level (black line) in the last column indicates 0

At 12 months, the primary analysis yielded a mean HbA1c of 6.5% (SD 1.3) in the intervention and 7.1% (SD 1.9) in the control arm (aMD −0.46%; 95%CI −1.14 to 0.22), with consistent results across all sensitivity analyses (Fig. 2).

Interaction p-values for the pre-specified potential effect modifiers on the primary endpoint were all larger than 0.1 (Additional File 3: Fig. S2).

**Fig. 3:**
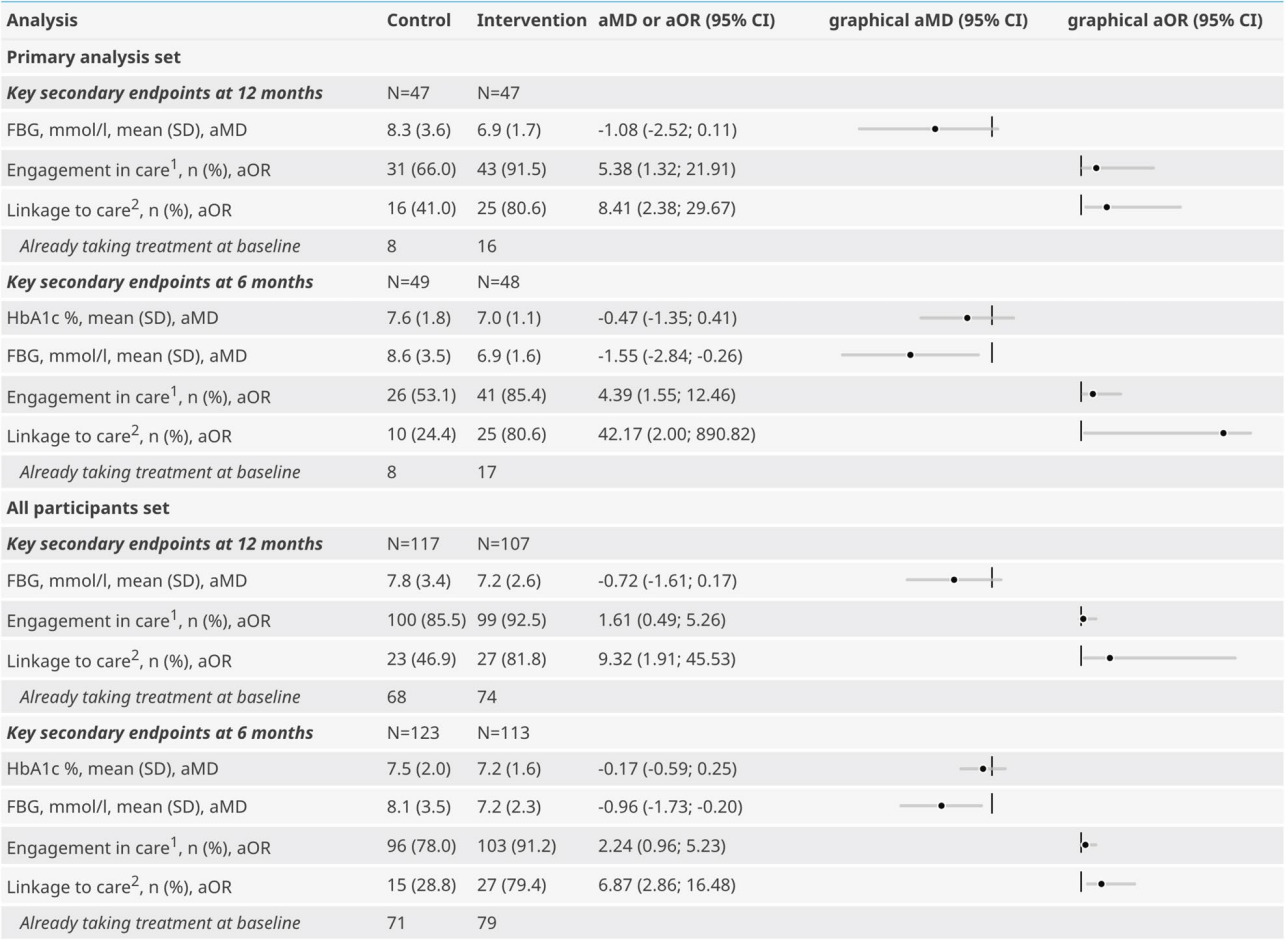
Key secondary endpoints, in primary analysis set and all participants set. 1: Engagement in care defined as reporting intake of antidiabetic medication or reaching control target without medication. Participants with missing information were considered not being engaged in care. 2: Linkage to care defined as having initiated antidiabetic treatment since enrolment among those not already taking antidiabetic treatment at baseline. The upper 95%CI in the last column was capped 50 for the estimate ‘Linkage to care’ at 6 months in the primary analysis set. The reference level (black line) in the second last column indicates 0, while in the last column it indicates 1. Abbreviations: FBG: fasting blood glucose; aOR: adjusted odds ratio; aMD: adjusted mean difference

Key secondary outcomes are summarized in Fig. 3. In the primary analysis set, at 12 months, FBG was 6.9 mmol/l (SD 1.7) in the intervention arm and 8.3 (SD 3.6) in the control arm (aMD −1.08; 95%CI −2.52 to 0.11). Engagement in care was 91.5% versus 66.0% (aOR 5.38; 95%CI 1.32 to 21.91), and linkage to care was 80.6% versus 41.0% (aOR 8.41; 95%CI 2.38 to 29.67) across the intervention and control arms, respectively. The same effect directions were observed at 6 months. In the all participants set, at 12 months, 99/107 (92.5%) were engaged in care in the intervention arm and 100/117 (85.5%) in the control arm (aOR 1.61; 95%CI 0.49 to 5.26).

Further secondary endpoints are presented in the Additional File 4: Tables S3 and S4.

**Fig. 4:**
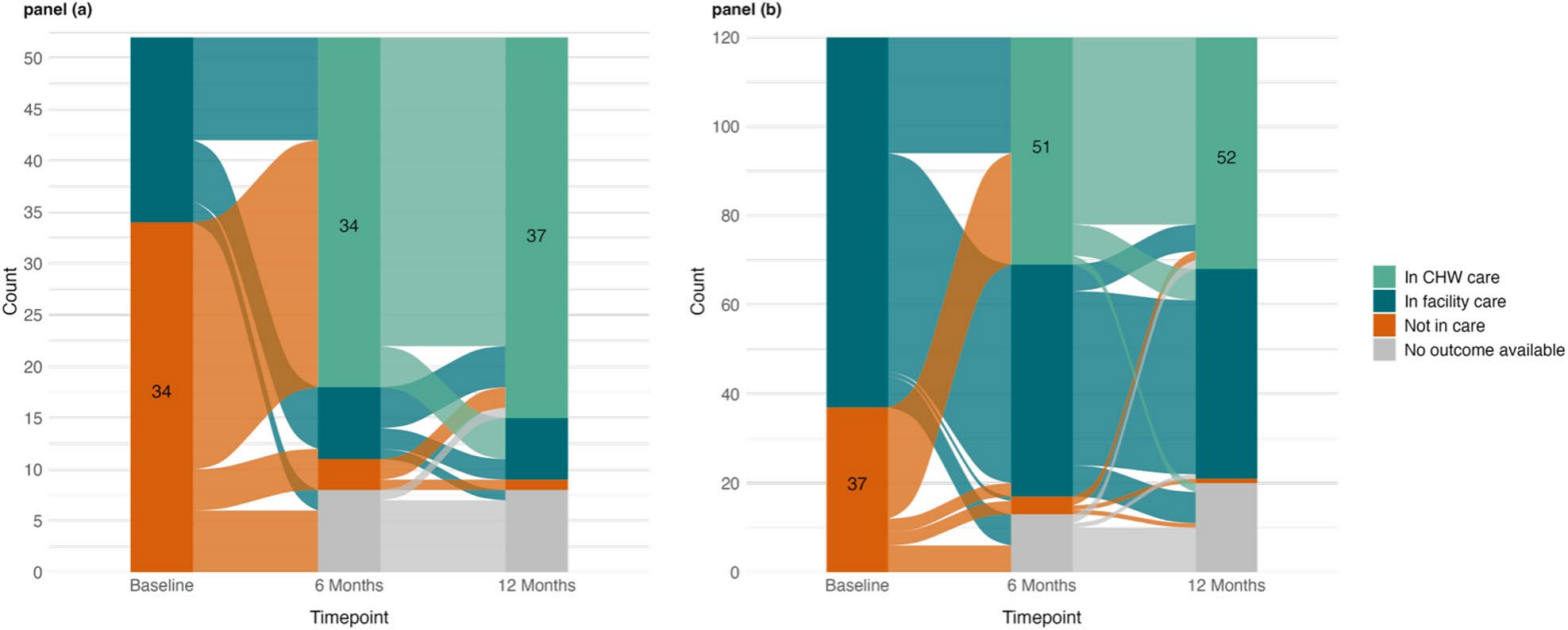
Trajectory of care, in the intervention arm, (**a**) primary analysis set, (**b**) all participants set. Abbreviations: CHW Community Health Worker

Figure 4 illustrates the trajectory of engagement in care in the intervention arm. Across all intervention participants (panel b), most people newly diagnosed at baseline were eligible for and chose CHW care and once CHW chosen, stayed with their CHW. Approximately half the people already in facility care at baseline were eligible for and chose CHW care. Of the 99 participants engaged in care at 12 months, 52/99 (52.5%) were in CHW care, meaning they were independently managed by their CHWs.

The proportion of participants being eligible for and choosing CHW care was higher in the primary analysis set (panel a), because participants with complicated diabetes (using insulin or more than one oral antidiabetic at baseline) who were often not eligible for CHW care were excluded from the primary analysis set. At 12 months, 37 of 43 engaged in care participants (86.0%) in the intervention arm of the primary analysis set were independently managed by their CHWs.

Across all participants, within 12 months, seven participants died in the intervention arm and six in the control arm (Table 2), two of each were part of the primary analysis set. Six and seven non-fatal serious adverse events occurred in the intervention and control arm across all participants, with two of them occurring in each arm among participants included in the primary analysis set. 28 and 31 adverse events of special interest were observed in the intervention and control arm in the all participants set, most of them related to hyperglycaemic events. Medication-related adverse events were not observed.

**Table 2:** Safety endpoints at 12 months

	Control	Intervention
**Primary analysis set**	N = 51	N = 52
Deaths, n (%)	2 (3.9)	2 (3.8)
Non-fatal serious adverse events, n (%)	2 (3.9)	2 (3.8)
*Hospitalization*	*2* (*3.9)*	*2* (*3.8)*
Adverse events of special interest^a^, n (%)	9 (17.6)	6 (11.5)
*Hyperglycaemic events, n (%)*	*9*	*7*
*New onset diabetic foot, n (%)*	*0*	*1*
**All participants set**	N = 132	N = 120
Deaths, n (%)	6 (4.5)	7 (5.8)
Non-fatal serious adverse events, n (%)	6 (4.5)	7 (5.8)
*Hospitalizations*	*6*	*6*
*Life-threatening event*		*1*
Adverse events of special interest^a^, n (%)	31 (23.5)	26 (21.7)
*Hyperglycaemic events, n (%)*	*29*	*23*
*New onset vision impairment, n (%)*	*1*	*1*
*New onset heart failure, n (%)*	*1*	*1*
*New onset diabetic foot, n (%)*	*0*	*1*

^a^Adverse events of special interest defined as as adverse events consistent with T2D complications, such as stroke, myocardial infarction, hyperglycaemic events (RBG > 16.7 mmol/l or FBG > 14.0 mmol/l or presence of all three cardinal symptoms of diabetes), new diagnosis of heart failure, chronic kidney disease, blindness, diabetic foot syndrome and adverse events probably related to intake of antidiabetic medication, such as significant hypoglycaemia (BG < 3 mmol/l and symptoms of hypoglycaemia) and side effects leading to discontinuation of the medication concerned

## Discussion

This trial provides the first randomized evidence suggesting that trained CHWs, guided by a CDSS application, may be able to safely and effectively initiate and monitor first-line pharmacotherapy for type 2 diabetes in a rural low-resource setting. Compared with facility-based care, the intervention was associated with higher linkage and engagement in care and a trend toward lower glycaemic levels. Effect estimates were directionally consistent across sensitivity analyses and aligned with findings from a sister trial in the same setting focused on hypertension [21]. However, the results should be interpreted cautiously, as the confidence interval around the effect estimate of the primary outcome was wide, reflecting substantial uncertainty due to the smaller-than-anticipated sample size. The results are therefore compatible with both clinically meaningful benefit and no effect.

Although modest, the observed reduction in HbA1c (−0.46%) is within the range considered clinically relevant in diabetes trials [40]. Reductions of this magnitude have been associated with meaningful shifts in glycaemic control and, when sustained, with reductions in microvascular complications [41]. While our study was not powered to assess clinical outcomes, these considerations suggest that even small differences in HbA1c may be relevant at the population level.

One of the key findings of our study was the substantial improvement in linkage to and engagement in care observed in the intervention arm. Participants receiving CHW-led care were markedly more likely to initiate antidiabetic treatment and remain engaged in care compared with those referred to facility-based services. In settings where access to facility-based chronic disease care is limited, improving linkage and sustained engagement may represent a critical mechanism through which community-based care models improve long-term outcomes. These findings suggest that the primary benefit of CHW-led diabetes care may lie in improving access to and continuity of care, with improved glycaemic control emerging as a downstream effect.

Several narrative reviews, pooling studies from low- and middle-incomes, highlighted the high acceptability of CHW-led diabetes interventions and their effectiveness in improving health knowledge and medication adherence [12–14, 42]. One recent scoping review [29] and two systematic reviews [14, 15] summarized the evidence on task-sharing diabetes care to CHW on diabetes outcomes, including HbA1c control. The scoping review suggested that community models could improve diabetes care, but highlighted the low quality of existing, mainly non-randomized, studies [29]. One systematic review excluded studies from low- and middle-income countries in the meta-analysis due to low quality and high heterogeneity [15], while the other one showed no reduction in HbA1c when care was delivered by CHWs [14]. None of the included trials assessed an intervention whereby trained CHWs independently initiate, monitor and titrate first-line pharmacotherapy for diabetes.

Many countries in southern Africa and other low- and middle-income countries have established CHW systems that are traditionally focusing on maternal and neonatal health and on communicable diseases, especially HIV/AIDS [43]. In recent years, increasing evidence has emerged showing a beneficial effect and high cost-effectiveness of CHW-led interventions for diseases outside the traditional scope, especially for NCDs [12, 13, 21, 42, 44–46]. Considering the increasing number of people requiring diabetes care and the limited professional healthcare workforce in low- and middle-income countries, the need for evidence-based interventions that can be implemented within existing CHW systems to effectively improve diabetes care outcomes while reducing the workload of healthcare professionals is evident and a key public health priority [6].

At twelve months, more than 80% of participants in care with uncomplicated, uncontrolled diabetes at baseline and more than 50% of all participants in care were independently managed by their CHW at the community level. These findings suggest a high level of trust and acceptability of the CHWs in their communities as well as the utility of the tablet-based CDSS tool to support the CHWs in their patient management. CDSS applications seem to emerge as a key enabler for the shifting of more complex tasks - including medication prescription - to CHWs [47, 48].

Safety is a key consideration when expanding the clinical responsibilities of CHWs to include pharmacological treatment. In our study, deaths and serious adverse events occurred at similar frequencies in both study arms, and medication-related adverse events were not observed. Hyperglycaemic events were numerically lower in the intervention arm, consistent with lower glycaemic levels observed among participants receiving CHW-led care. While the small sample size limits definitive conclusions regarding safety, these findings provide preliminary evidence suggesting that CHWs supported by a clinical decision support system may be able to safely initiate and monitor first-line diabetes pharmacotherapy in rural settings.

This study has several strengths. First, our intervention is pragmatic and generalizable to other settings with a similar CHW programme. The intervention was fully integrated within the routine government health system in Lesotho. Task-shifting to CHWs was facilitated by an open-source software framework that is widely used in CHW programmes across low- and middle-income countries and interoperable with most data management systems [36]. In addition to providing clinical decision support, the framework facilitates community-level data collection, which has been identified as a major bottleneck for strengthening community health systems [49]. Second, we chose a comprehensive approach towards cardiovascular risk management for diabetes patients, as recommended by international treatment guidelines. Cardiovascular diseases are the main cause of death among people living with diabetes but their comprehensive management are often poorly implemented in clinical routine as highlighted by the low number of participants in our trial on lipid-lowering medication at baseline. Thanks to the integration of statin prescription in our intervention, the proportion of participants accessing lipid-lowering medication increased substantially. In our trial, CHWs not only delivered diabetes care, but a similar care package for people with hypertension, designed and reported as a sister trial [21]. Third, besides providing a comprehensive cardiovascular care package, the CHWs also regularly collected sociodemographic and health-related data in their villages as part of the ComBaCaL cohort assessments [23]. This automatically ensured completeness of follow-up data and high-quality data on safety. No apparent difference in safety endpoints was observed between the arms.

Several limitations merit consideration. The most important is the underpowered sample size, driven by a lower-than-anticipated type 2 diabetes prevalence, stringent eligibility criteria for the primary analysis set, and being constrained by the size of our cohort. As a result, the confidence intervals around effect estimates are wide, and definitive conclusions cannot be drawn. Second, endpoint assessments were conducted by unmasked CHWs or study staff at the participants’ home. However, the primary endpoint (HbA1c) is unlikely to be susceptible to performance bias. Third, while the CHWs in our study were part of the government CHW system, they received 20$ per month in addition to the regular government stipend of 40$ to compensate the study-related workload. However, all CHWs in both arms received the same non-performance-based stipend, which compensated study-related workload and is therefore unlikely to contribute to between-group differences. Fourth, some baseline characteristics were imbalanced across study arms in the primary analysis set, including the proportion of participants already receiving diabetes care at baseline, which may have influenced outcome estimates. However, the analysis model was adjusted for baseline HbA1c which may partially account for differences in baseline disease severity and care engagement. Such imbalance was not observed in the larger all participants set. Fifth, cause-of-death information was not systematically available, as most deaths occurred outside health facilities and were reported through community informants. The number of deaths was similar between study arms. Finally, the empirical ICCs observed in the primary analysis were higher than anticipated. This likely reflects the very small number of participants per cluster in the primary analysis set (median one participant per village), which is known to lead to unstable ICC estimates in cluster-randomized trials. With such sparse cluster sizes, ICC estimates are particularly sensitive to random variation and should therefore be interpreted cautiously.

Despite these caveats, the findings suggest that trained CHWs may be able to extend their scope to include first-line pharmacological diabetes management when supported by robust digital tools, with potential benefits for care delivery in resource-constrained settings. Given the rapidly rising diabetes burden in low- and middle-income countries, further investigation of digitally supported task-shifting models within existing community health systems is warranted.

## Conclusions

This cluster-randomized trial provides preliminary evidence suggesting that trained CHWs, supported by a digital clinical decision support system, may be able to safely deliver first-line pharmacological care for type 2 diabetes in a rural low-resource setting. Compared with facility-based care, the CHW-led model showed consistently higher linkage and engagement in care and a trend toward lower glycaemic levels without apparent safety concerns. Although the trial was underpowered to draw definitive conclusions, the direction and consistency of effects across analyses support the potential of digitally supported task-shifting for diabetes care. Larger, adequately powered trials with longer follow-up are needed to confirm effectiveness and to inform sustainable integration of CHW-led diabetes management into routine health systems in low- and middle-income countries.

## Supplementary information

Supplementary information 1: Additional File 1 Table S1.CONSORT checklist – CRT extension

Supplementary information 2: Additional File 2 Figure S1. Treatment algorithm for type 2 diabetes management provided by community health workers

Supplementary information 3: Additional File 3 Figure S2. Forestplot of potential effect modifiers on primary endpoint

Supplementary information 4: Additional File 4 Table S2. Further secondary endpoints, in primary analysis set. **Table S3**. Further secondary endpoints, in all participants set

## Data Availability

A de-identified dataset, the statistical report and analysis code are available on Zenodo [50]. The trial was registered with ClinicalTrials.gov (NCT05743387), where a full protocol and statistical analysis plan are available. A study protocol manuscript has been published previously [31]. Requests for access to more detailed data may be made to the corresponding author by submitting a proposal, which will be reviewed by the trial consortium (www.combacal.org). The code for the CDSS application used by CHWs for data collection and service delivery can be found at https://github.com/clinepi-usb/cht-combacal. Access to the test environment of the application is available from the corresponding authors upon reasonable request.

## Abbreviations

aMD: Adjusted mean difference
aOR: Adjusted odds ratio
BG: Blood glucose
BMI: Body mass index
CDSS: Clinical decision support system
CHT: Community Health Toolkit
CHW: Community health worker
CI: Confidence interval
ComBaCaL: Community-Based Chronic Care Lesotho
CONSORT: Consolidated Standards of Reporting Trials
CVD: Cardiovascular disease
FBG: Fasting blood glucose
HbA1c: Glycated hemoglobin
HDL: High-density lipoprotein
HIV: Human immunodeficiency virus
IPAQ-SF: International Physical Activity Questionnaire Short Form
IQR: Interquartile range
LDL: Low-density lipoprotein
NCD: Non-communicable disease
RBG: Random blood glucose
SD: Standard deviation
TwiCs: Trials within Cohorts
WHO: World Health Organization
