# Supplementary material for: Community health worker-led versus facility-based type 2 diabetes care in rural Lesotho: a cluster-randomized trial within the ComBaCaL cohort study
Source: BMC Med. 2026 May 22;24:400. doi: 10.1186/s12916-026-04943-4 (PMC13374172; doi:10.1186/s12916-026-04943-4)
Supplement: Supplementary file 2 — Supplementary information 2: Additional File 2 Figure S1. Treatment algorithm for type 2 diabetes management provided by community health workers [file 12916_2026_4943_MOESM2_ESM.docx]

## Additional File 2

# Figure S1. Treatment algorithm for type 2 diabetes management provided by community health workers


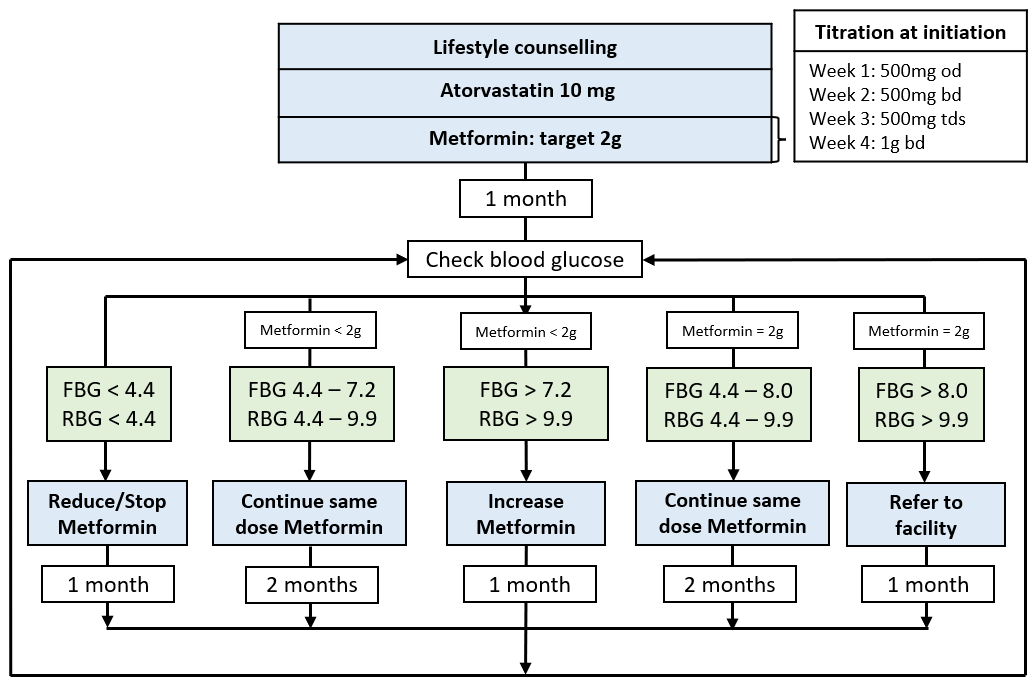


**Figure S1 legend**

FBG: fasting blood glucose; RBG: random blood glucose
