# Supplementary material for: Community health worker-led versus facility-based type 2 diabetes care in rural Lesotho: a cluster-randomized trial within the ComBaCaL cohort study
Source: BMC Med. 2026 May 22;24:400. doi: 10.1186/s12916-026-04943-4 (PMC13374172; doi:10.1186/s12916-026-04943-4)
Supplement: Supplementary file 3 — Supplementary information 3: Additional File 3 Figure S2. Forestplot of potential effect modifiers on primary endpoint [file 12916_2026_4943_MOESM3_ESM.docx]

## Additional File 3

# Figure S2. Forestplot of potential effect modifiers on primary endpoint

**Figure S2 legend**

Age was analysed as continuous interaction, assuming linearity
