# Supplementary material for: Community health worker-led versus facility-based type 2 diabetes care in rural Lesotho: a cluster-randomized trial within the ComBaCaL cohort study
Source: BMC Med. 2026 May 22;24:400. doi: 10.1186/s12916-026-04943-4 (PMC13374172; doi:10.1186/s12916-026-04943-4)
Supplement: Supplementary file 4 — Supplementary information 4: Additional File 4 Table S2. Further secondary endpoints, in primary analysis set. Table S3. Further secondary endpoints, in all participants set [file 12916_2026_4943_MOESM4_ESM.docx]

## Additional File 4

# Table S2. Further secondary endpoints in the primary analysis set

|  | **Control** | **Intervention** | **aOR or aMD (95% CIs)** |
| --- | --- | --- | --- |
| **Secondary endpoints at 6 months^1^** | N=49 | N=48 |  |
| Self-reported full adherence to antidiabetic medication^2^, n (%) | 14 (100.0) | 38 (97.4) | Not estimable |
| Not on treatment | 34 | 9 |  |
| WHO CVD risk ≥10%^3^, n (%) | 16 (44.4) | 28 (70.0) | 1.06 (0.18; 6.13) |
| Current statin use, n (%) | 2 (4.3) | 26 (59.1) | 77.3 (8.22; 726.99) |
| Total cholesterol, mg/dL, mean (SD) | 150.9 (32.6) | 153.6 (40.8) | -4.37 (-19.06; 10.32) |
| LDL cholesterol, mg/dL, mean (SD) | 82.9 (28.9) | 91.5 (35.1) | 0.84 (-14.73; 16.4) |
| BMI, kg/m^2^, mean (SD) | 27.9 (5.8) | 30.0 (6.0) | 0.05 (-0.86; 0.96) |
| Abdominal circumference, cm, mean (SD) | 94.4 (13.4) | 96.1 (13.9) | 0.69 (-2.95; 4.33) |
| Moderate/high physical activity^4^, n (%) | 38 (79.2) | 36 (83.7) | 6.43 (0.02; 1729.57) |
| Current smoking, n (%) | 13 (27.7) | 12 (27.3) | 0.81 (0.24; 2.80) |
| Alcohol consumption ≥1 day/week, n (%) | 6 (12.4) | 5 (12.2) | 2.18 (0.43; 11.0) |
| **Secondary endpoints at 12 months^1^** | N=47 | N=47 |  |
| Self-reported full adherence to antidiabetic medication^2^, n (%) | 21 (100.0) | 33 (89.2) | Not estimable |
| Not on treatment | 24 | 10 |  |
| WHO CVD risk ≥10%^3^, n (%) | 21 (50.0) | 28 (73.7) | 8.15 (0.31; 212.39) |
| Current statin use, n (%) | 5 (11.1) | 27 (61.4) | 12.88 (4.01; 41.39) |
| Total cholesterol, mg/dL, mean (SD) | 152.9 (39.3) | 154.5 (41.5) | -0.46 (-20.59; 19.68) |
| LDL cholesterol, mg/dL, mean (SD) | 86.1 (33.8) | 91.0 (31.8) | -1.98 (-18.5; 14.54) |
| BMI, kg/m^2^, mean (SD) | 27.8 (5.6) | 30.2 (5.7) | 0.02 (-0.91; 0.96) |
| Abdominal circumference, cm, mean (SD) | 95.4 (12.8) | 95.7 (12.6) | -0.67 (-3.86; 2.53) |
| Moderate/high physical activity^4^, n (%) | 31 (70.5) | 31 (72.1) | 1.51 (0.34; 6.63) |
| Current smoking, n (%) | 10 (22.2) | 10 (22.7) | 0.68 (0.12; 3.78) |
| Alcohol consumption ≥1 day/week, n (%) | 9 (20.9) | 2 (4.6) | 0.32 (0.04; 2.84) |

**Table S2 legend**

1: Same intercurrent event handling as for primary analysis

2: Full adherence defined as reporting intake of antidiabetic medication on all of the four last days

3: 10-year risk for a fatal or non-fatal cardiovascular event estimated using the lab-based WHO cardiovascular disease risk prediction tool

4: Self-reported physical activity using the International Physical Activity Questionnaire Short Form (IPAQ-SF)

Abbreviations: LDL: Low-density lipoprotein, BMI: body-mass index; aOR: adjusted odds ratio; aMD: adjusted mean difference

Missing data:

Adherence: 1 at 6 months (1 in control) and 2 at 12 months (2 in control)

WHO CVD risk: 21 at 6 months (13 in control, 8 in intervention) and 14 at 12 months (5 in control, 9 in intervention)

Current statin use: 7 at 6 months (3 in control, 4 in intervention) and 5 at 12 months (2 in control, 3 in intervention)

Total cholesterol: 8 at 6 months (3 control, 5 intervention) and 6 at 12 months (3 control, 3 intervention)

LDL: 27 at 6 months (17 control, 10 intervention) and 18 at 12 months (10 control, 8 intervention)

BMI: 8 at 6 months (4 control, 4 intervention) and 6 at 12 months (2 control, 4 intervention)

Abdominal circumference: 7 at 6 months (2 control, 5 intervention) and 9 at 12 months (5 control, 4 intervention)

Physical activity: 6 at 6 months (1 control, 5 intervention) and 7 at 12 months (3 control, 4 intervention)

Smoking: 6 at 6 months (2 control, 4 intervention) and 5 at 12 months (2 control, 3 intervention)

Alcohol consumption: 8 at 6 months (4 control, 4 intervention) and 8 at 12 months (4 control, 4 intervention)

# Table S3. Further secondary endpoints in the all participants set

|  | **Control** | **Intervention** | **aOR or aMD (95% CIs)** |
| --- | --- | --- | --- |
| **Secondary endpoints at 6 months^1^** | N=123 | N=113 |  |
| Self-reported full adherence to antidiabetic medication^2^, n (%) | 75 (92.6) | 90 (92.8) | 1.08 (0.33; 3.59) |
| Not on treatment | 42 | 16 |  |
| WHO CVD risk ≥10%^3^, n (%) | 51 (51.0) | 66 (71.7) | 1.18 (0.44; 3.13) |
| Current statin use, n (%) | 6 (5.1) | 45 (42.5) | 13.62 (5.44; 34.12) |
| Total cholesterol, mg/dL, mean (SD) | 152.7 (34.5) | 154.5 (42.9) | -0.77 (-12.11; 10.57) |
| LDL cholesterol, mg/dL, mean (SD) | 88.3 (31.9) | 85.6 (32.7) | -7.59 (-18.26; 3.08) |
| BMI, kg/m^2^, mean (SD) | 28.2 (5.6) | 29.5 (5.8) | 0.27 (-0.39; 0.93) |
| Abdominal circumference, cm, mean (SD) | 95.4 (13.5) | 95.5 (13.5) | 0.60 (-1.54; 2.74) |
| Moderate/high physical activity^4^, n (%) | 92 (76.7) | 82 (78.1) | 1.27 (0.44; 3.67) |
| Current smoking, n (%) | 23 (19.3) | 25 (23.6) | 1.43 (0.49; 4.12) |
| Alcohol consumption ≥1 day/week, n (%) | 11 (9.2) | 10 (9.9) | 1.14 (0.44; 2.95) |
| **Secondary endpoints at 12 months^1^** | N=117 | N=107 |  |
| Self-reported full adherence to antidiabetic medication^2^, n (%) | 83 (94.3) | 83 (89.2) | 0.38 (0.10; 1.52) |
| Not on treatment | 29 | 14 |  |
| WHO CVD risk ≥10%^3^, n (%) | 61 (56.0) | 66 (74.2) | 3.36 (0.99; 11.36) |
| Current statin use, n (%) | 16 (13.9) | 54 (54.0) | 7.45 (3.78; 14.71) |
| Total cholesterol, mg/dL, mean (SD) | 160.6 (40.1) | 158.2 (41.6) | -3.23 (-15.37; 8.90) |
| LDL cholesterol, mg/dL, mean (SD) | 92.7 (32.8) | 88.0 (36.4) | -6.87 (-18.45; 4.71) |
| BMI, kg/m^2^, mean (SD) | 28.5 (5.7) | 29.6 (5.6) | 0.21 (-0.51; 0.93) |
| Abdominal circumference, cm, mean (SD) | 96.3 (13.0) | 95.6 (13.0) | -0.85 (-3.12; 1.42) |
| Moderate/high physical activity^4^, n (%) | 80 (70.2) | 73 (73.7) | 1.78 (0.48; 6.68) |
| Current smoking, n (%) | 17 (14.8) | 27 (27.0) | 3.46 (1.17; 10.24) |
| Alcohol consumption ≥1 day/week, n (%) | 13 (12.6) | 5 (5.1) | 0.53 (0.14; 2.03) |

**Table S3 legend**

1: Same intercurrent event handling as for primary analysis

2: Full adherence defined as reporting intake of antidiabetic medication on all of the four last days

3: 10-year risk for a fatal or non-fatal cardiovascular event estimated using the lab-based WHO cardiovascular disease risk prediction tool

4: Self-reported physical activity using the International Physical Activity Questionnaire Short Form (IPAQ-SF)

Abbreviations: LDL: Low-density lipoprotein, BMI: body-mass index; aOR: adjusted odds ratio; aMD: adjusted mean difference

Missing data:

Adherence: 4 at 6 months (2 in control, 2 in intervention) and 2 at 12 months (2 in control)

WHO CVD risk: 44 at 6 months (23 in control, 21 in intervention) and 26 at 12 months (8 in control, 18 in intervention)

Current statin use: 12 at 6 months (5 in control, 7 in intervention) and 9 at 12 months (2 in control, 7 in intervention)

Total cholesterol: 14 at 6 months (5 control, 9 intervention) and 12 at 12 months (3 control, 9 intervention)

LDL: 49 at 6 months (25 control, 24 intervention) and 35 at 12 months (17 control, 18 intervention)

BMI: 23 at 6 months (13 control, 10 intervention) and 12 at 12 months (4 control, 8 intervention)

Abdominal circumference: 19 at 6 months (9 control, 10 intervention) and 18 at 12 months (8 control, 10 intervention)

Physical activity: 11 at 6 months (3 control, 8 intervention) and 11 at 12 months (3 control, 8 intervention)

Smoking: 11 at 6 months (4 control, 7 intervention) and 9 at 12 months (2 control, 7 intervention)

Alcohol consumption: 16 at 6 months (4 control, 12 intervention) and 13 at 12 months (5 control, 9 intervention)
